# Evaluation of the efficacy and safety of rivaroxaban compared to warfarin in patients with left ventricular apical thrombus: a randomized clinical trial

**DOI:** 10.1186/s12959-024-00632-5

**Published:** 2024-07-19

**Authors:** Pejman Mansouri, Zahra Azamian Jazi, Mohammad Hadi Mansouri, Hooman Dehghan, Reihaneh Zavar, Seyedeh Melika Hashemi, Fereshteh Sattar, Masoumeh Sadeghi, Afshin Amirpour, Morteza Abdar

**Affiliations:** 1 Hypertension Research Center, Cardiovascular Research Institute, Isfahan University of Medical Sciences, Isfahan, Iran; 2 Isfahan Cardiovascular Research Center, Cardiovascular Research Institute, Isfahan University of Medical Sciences, Isfahan, Iran; 3 Tehran Heart Center, Tehran University of Medical Sciences, Tehran, Iran; 4 Cardiac rehabilitation research center, cardiovascular research institute, Isfahan University of Medical Sciences, Isfahan, Iran

**Keywords:** Left ventricular apical thrombus, Rivaroxaban, Warfarin, ACS

## Abstract

**Introduction:**

This research is one of the pioneering randomized clinical trials (RCTs) aimed at assessing the effectiveness and safety of rivaroxaban in treating left ventricular thrombus (LVT) in patients who have experienced acute coronary syndrome (ACS).

**Materials and methods:**

This is a randomized, controlled, interventional, open-label study. The patients were randomly divided into warfarin and rivaroxaban groups. We performed transthoracic echocardiography at the start of the study and again after three months to measure the thrombus area in square millimeters. The morphology of the thrombus was categorized into mural and round, and the mobility was classified into immobile, semi-mobile and hypermobile. We also monitored for adverse events including bleeding, systemic embolic occurrences, rehospitalization, and major adverse cardiac events (MACE).

**Results:**

The study included fifty-two patients in the intention-to-treat analysis, with an equal split between the rivaroxaban and warfarin groups (26 patients each). The average follow-up duration was three months. The thrombus resolution rates in the rivaroxaban (76.9%) and warfarin (69.2%) groups, as well as the thrombus size reduction, did not show statistical significance between groups. All semi-mobile or hypermobile thrombi transformed into immobile and all of the round LVTs changed into a mural in both rivaroxaban and warfarin groups. No significant difference was observed in bleeding complications and rehospitalization between the two groups.

**Conclusion:**

The trial demonstrated that rivaroxaban is as effective as warfarin in terms of thrombus resolution rate, reduction in thrombus size, bleeding risk, and rehospitalization rate. Our findings suggest that rivaroxaban is a viable alternative to warfarin for managing left ventricular thrombus.

**Graphical Abstract:**

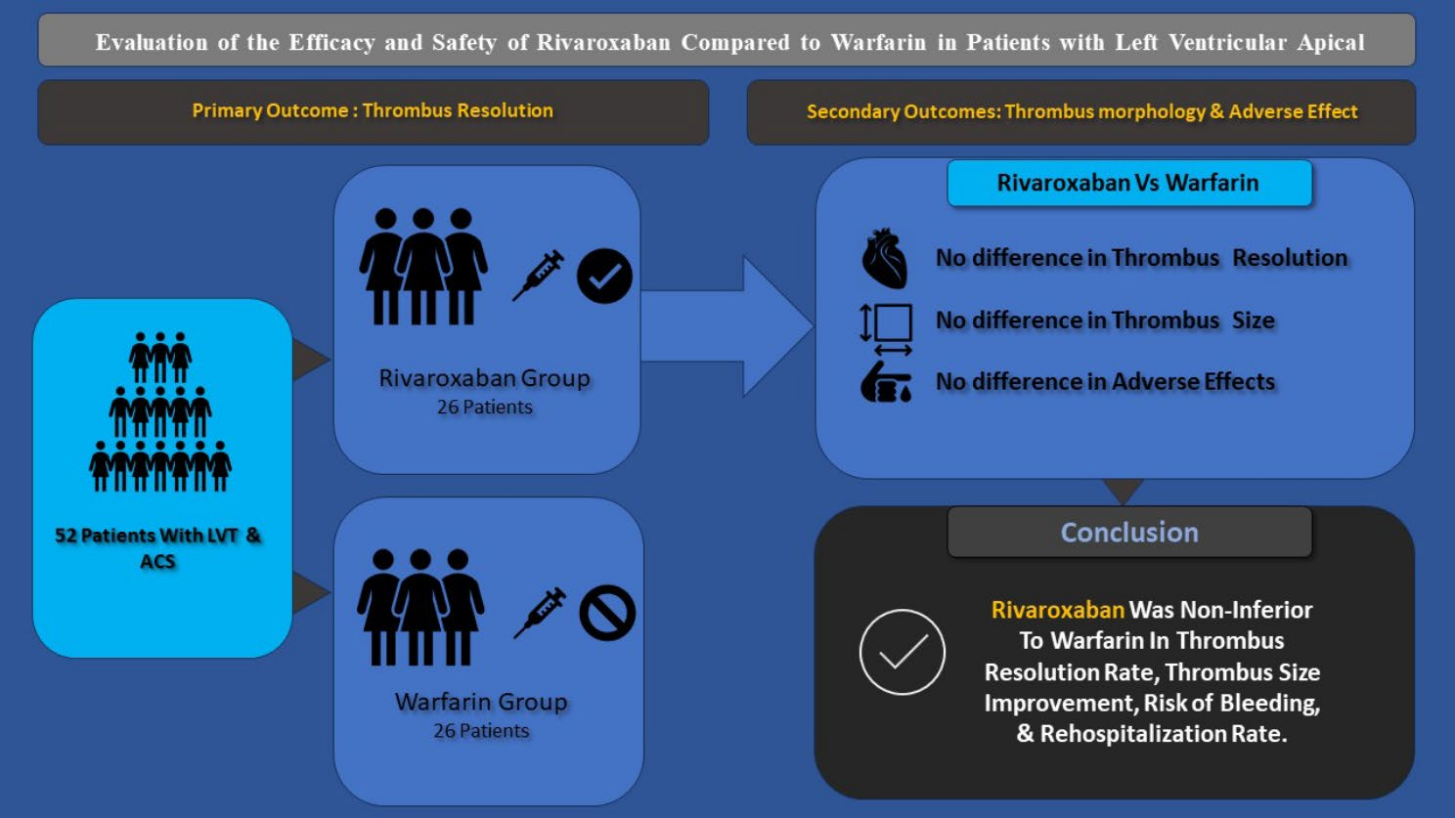

## Introduction

Left ventricular thrombosis (LVT) is a potentially life-threatening event that mainly occurs in patients with left ventricular systolic dysfunction, especially 1–14 days after myocardial infarction (MI) [1–3]. LVT formation is significantly associated with an increased risk of MI, stroke, distal embolization, other major cardiovascular events due to systemic thromboembolism, and death. This risk has been reported to be about 10–40%^[4, 4^. Although the prevalence of LVT has decreased compared to the pre-thrombolytic era, it has been reported to be 2–7% in patients with severe left ventricular dysfunction and could rise to 7–22% in patients with ST-elevation myocardial infarction (STEMI) [5]. Vitamin K antagonists (VKAs) such as Warfarin are approved by the Food and Drug Administration (FDA) as first-line and standard anticoagulant therapy for the management of LVT with the international normalized ratio (INR) 2–3 during the treatment period [6].

Rivaroxaban, a direct factor Xa inhibitor, belongs to the class of Direct Oral Anticoagulants (DOACs). It has received regulatory approval for various clinical indications, including approval for the treatment of deep venous thrombosis, pulmonary embolism, and non-valvular atrial fibrillation. One notable aspect of Rivaroxaban is its use for the prevention of atherothrombotic events following acute coronary syndrome (ACS) [7].

Additionally, DOACs such as rivaroxaban are emerging treatments for LVT with fewer drug and dietary interactions, a reduced risk of intracranial hemorrhage, no need for routine monitoring, and enhanced drug adherence compared to VKAs [8,9]. The assessment of the safety and efficacy of DOACs in the management of LVT in patients with acute coronary syndrome (ACS) has been limited to heterogeneous retrospective observational studies [10,11]. To our knowledge, there is only one randomized clinical trial (RCT) investigating the efficacy and safety of rivaroxaban compared to warfarin in patients with LVT [12]; however, it does not specifically focus on ACS patients. Our study is one of the first RCTs to evaluate the efficacy and safety of rivaroxaban in the management of LVT in ACS patients.

## Materials and methods

### Study design

This randomized, controlled, interventional, open-label non-inferiority trial was conducted at the Chamran Cardiovascular Medical and Research Center, a tertiary referral center affiliated with Isfahan University of Medical Sciences. The Ethics Committee approved the study protocol based on code number " IR.MUI.MED.REC.1399.710”. Furthermore, it has been registered in the Iranian Registry of Clinical Trials (IRCT20210614051574N9,02/19/2022). The study protocol was explained to the patients and their families, and they were reassured regarding the confidentiality of their personal information; they then signed written informed consent.

### Participants

The inclusion criteria encompassed adult patients (> 18 years old) admitted with a diagnosis of ACS, undergoing percutaneous coronary intervention (PCI), and having LV apical thrombus detected in their echocardiography results. Exclusion criteria consisted of, eGFR < 30 ml/min per 1.73 m [2], concomitant major trauma or active bleeding, uncontrolled hypertension, prior hemorrhagic stroke, history of major ischemic stroke, current oral anticoagulation for other reasons such as AF and VTE, active liver disease (regardless of its etiology), pregnancy, and inherited or acquired bleeding disorders.

### Intervention

Eligible patients were randomly allocated in a 1:1 ratio, to receive either Warfarin 5 mg daily or rivaroxaban 20 mg daily. The randomization was performed using a random number table, generated by a computerized electronic system. Those in the warfarin group received a loading dose of 5 mg orally, followed by a maintenance dose to maintain the INR between 2 and 3. For patients taking Warfarin, Time in Therapeutic Range (TTR) was calculated using the Rosendaal method [13]. The patients also received low-dose aspirin + clopidogrel as part of dual antiplatelet therapy (DAPT), alongside anticoagulant therapy. Triple therapy was continued for one month and then changed to dual therapy with clopidogrel and anticoagulant.

### Assessments and follow-up

The patient cohort comprised individuals who underwent a transthoracic echocardiographic examination upon admission and during a three-month follow-up period. The echocardiography utilized Two-Dimensional and Doppler echocardiographic examinations with a Philips EPIQ cardiac ultrasound machine, conducted by two experienced cardiologists with echocardiography fellowship training. Also, the data collection process was conducted by clinical research staff with appropriate training, such as research nurses or cardiology residents.

LV apical thrombus diagnosis was based on the identification of an echo-dense mass with clear margins adjacent to the myocardium and abnormal contractility in at least two echocardiographic views.

The greatest width (a vertical line from endocardium to thrombus-blood interface) and length of the thrombus were measured in the apical four-chamber view, and the thrombus area was calculated in mm [2]. The morphology of thrombus was categorized into mural and round. The border of a mural thrombus is primarily along with the endocardium and round thrombus is often spherical with protrusion to the LV cavity. The mobility of thrombus was classified into three categories; immobile, semi-mobile, and hypermobile. A mobile thrombus was defined by independent motion compared to the adjacent myocardium [14,15].

### Outcome measures

The primary outcome involved assessing thrombus resolution by TTE after a 3-month treatment follow-up. Secondary outcomes encompassed clinical measures (including adverse effects such as bleeding, systemic embolic events, rehospitalization, and major adverse cardiac events) as well as echocardiographic measures, focusing on changes in thrombus size, mobility, and morphology.

### Statistical analysis

Data were statistically analyzed using SPSS 25 for Windows (SPSS Inc., Chicago, IL, USA). Results are shown as mean ± standard deviation (SD) and as numbers and percentages for categorical data. Categorical variables were analyzed using the χ2 test, and continuous variables were analyzed using the Student’s t-test, and also non-parametric tests, including Chi-square-test, Wilcoxon Signed Ranks test, and Mann-Whitney test. P-value less than 0.05 was considered statistically significant.

## Results

### Study recruitment and follow-up

**Fig. 1:**
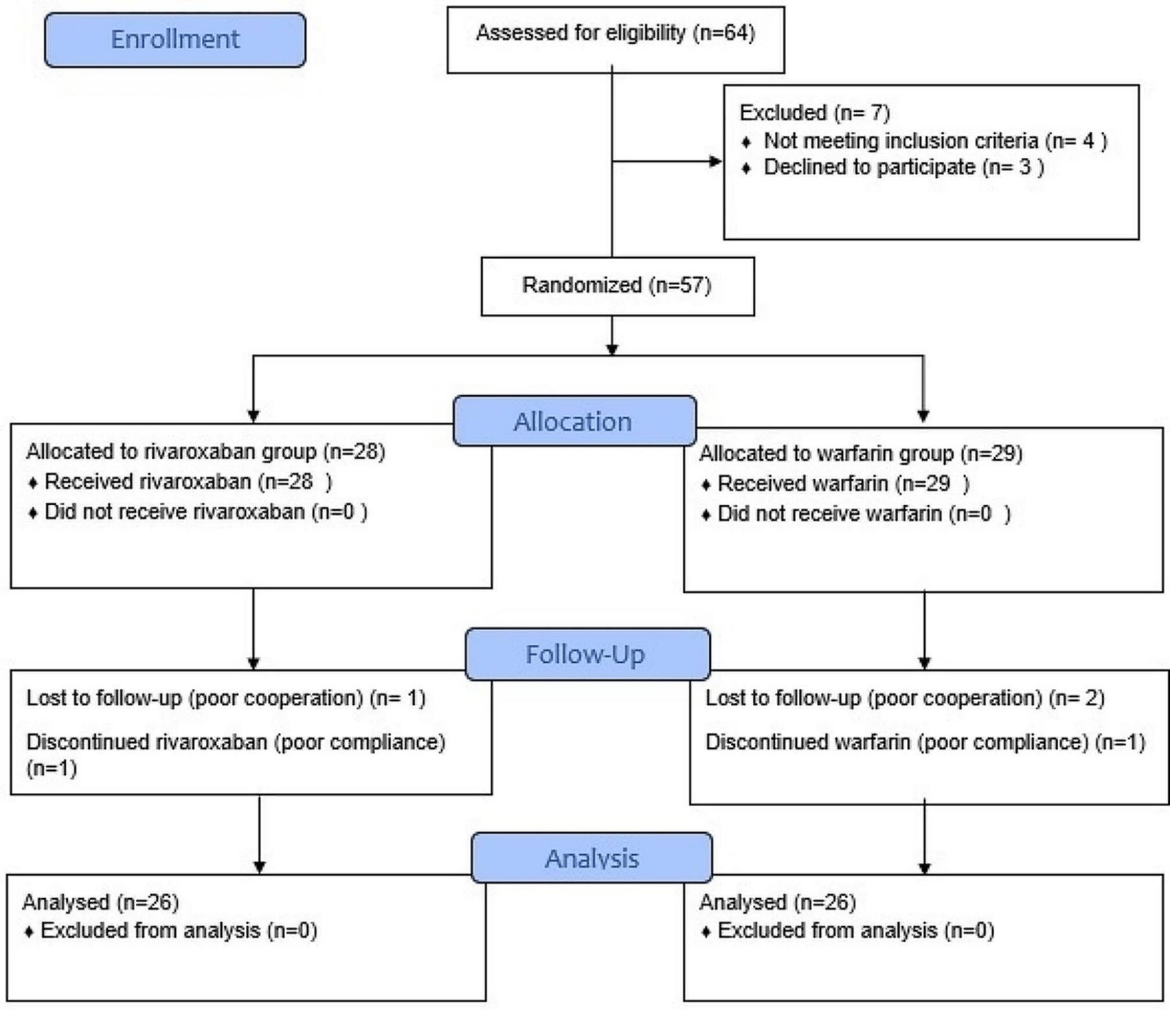
CONSORT flow diagram

A total of 57 patients underwent randomization in our study (Fig. 1. Trial Flow Diagram). However, 5 were lost to follow up, and 52 were included in the intention-to-treat analysis (26 in the rivaroxaban group and 26 in the warfarin group). The average follow-up period was three months.

Baseline patient characteristics.

Baseline characteristics of patients are summarized in Table 1. A total of 52 post-PCI patients with detected LVT were included. Both the rivaroxaban and warfarin groups were well matched in terms of age, sex, LVEF, and hemoglobin levels. The mean age was 56.50 ± 10.03 years, and the gender distribution was similar in both groups (88.5% and 80.8% male in the rivaroxaban and warfarin groups, respectively). The degree of LV impairment was similar in both groups, with a baseline LVEF of 29.58 ± 7.42 and 31.27 ± 8.29 in rivaroxaban and warfarin groups, respectively, with no statistically significant difference (p-value = 0.44).

**Table 1:** Baseline characteristics. Comparing between groups using independent samples t-test and crosstabs with Chi-square-test

Variable, Mean ±SD/ *N* (%)	Total (*N*=52)	Rivaroxaban (*N*=26)	Warfarin (*N*=26)	*p*-value
**Age (year)**	56.50$\pm$10.03	57.92$\pm$10.16	55.08$\pm$9.88	0.311
**Sex** Male Female	44 (84.6%) 8 (15.4%)	(88.5%) 23 3 (11.5%)	21 (80.8%) 5 (19.2%)	0.442
**Baseline LVEF**	30.42$\pm 7.84$	29.58$\pm 7.42$	31.27$\pm$8.29	0.442
**BMI (kg/m2)**	28.14±7.03	28.37±6.71	27.91±7.33	0.814
**DM**	23(44.2%)	11(42.3%)	12(46.1%)	0.854
**HTN**	19(36.5%)	10(38.4%)	9(34.6%)	0.863
**Smoker**	23(44.2%)	12(46.1%)	11(42.3%)	0.854
**Creatinine (mg/dl)**	1.19±0.27	1.17±0.18	1.21±0.35	0.606
**Hemoglobin(mg/dl)**	13.25±2.4	13.1±2.3	13.4±2.5	0.654
**Baseline Mobility** Immobile, N (%) Semimobile, N (%) hypermobile, N (%)	23 (44.2%) 20 (38.5%) 9 (17.3%)	12 (46.2%) 10 (38.5%) 4 (15.4%)	11 (42.3%) 10 (38.5%) 5 (19.2%)	0.926
**Baseline Morphology** Mural, N (%) Round, N (%)	35 (67.3%) 17 (32.7%)	18 (69.2%) 8 (30.8%)	17 (65.4%) 9 (34.6%)	0.768

LVEF: left ventricular ejection fraction, BMI: body mass index, DM: diabetes mellitus, HTN: hypertension

### Clinical outcomes

#### Thrombus resolution

During the 3-month follow-up, the overall rate of LV thrombus resolution was 73.1%. In the rivaroxaban group, 76.9% of patients experienced resolution, while in the warfarin group, 69.2% saw resolution. However, the comparison between the two groups did not yield statistical significance (p-value = 0.53), as depicted in Table 2.

**Table 2:** Comparison of thrombus resolution, bleeding and rehospitalization between groups using crosstabs with Chi-square-test

Variable	Total	Rivaroxaban	Warfarin	*p*-value
Thrombus resolution				
Yes, N(%)	38 (73.1)	20 (76.9)	18 (69.2)	0.532
No, N(%)	14 (26.9)	6 (23.1)	8 (30.8)	
**Bleeding**				
No, N(%)	50 (96.2)	25 (96.2)	25 (96.2)	1.000
Yes, N(%)	2 (3.8)	1 (3.8)	1 (3.8)	
**Rehospitalization**				
No, N(%)	49 (94.2)	25 (96.2)	24 (92.3)	0.552
Yes, N(%)	3 (5.8)	1 (3.8)	2 (7.7)	

Among patients taking Warfarin, 84.62% were within the therapeutic range (TTR ≥ 50%), while 15.38% were below the therapeutic range (TTR < 50%). Notably, patients with good control showed a trend toward greater thrombus resolution (81.8% resolution) compared to those with sub-optimal control (0% resolution) over the follow-up period (comparison between groups was conducted using Independent Samples t-test and crosstabs with Chi-square-test, p-value = 0.001).

### Risk of bleeding complications, rehospitalization, systemic embolic events and MACE

We found no difference in bleeding complications between these groups, the pooled risk ratio (RR) was 1, as shown in Table 2. Minor bleeding event happened in each group and the patients did not experience any major bleeding.

Overall information on the rehospitalization of patients is available in both groups (Table 2). The rate of rehospitalization was 3.8% and 7.7% in the rivaroxaban and warfarin groups, respectively. No statistically significant difference in rehospitalization rate was seen between the two treatment groups (p-value = 0.55). Decompensated heart failure (DHF) was the cause of rehospitalization in both groups. Also, no embolic events, stroke or MACE were detected in both groups.

### Echocardiographic measurement (thrombus size, morphology, and mobility)

Thrombus Characteristics were described in terms of size, morphology, and mobility based on echocardiographic images. Echocardiographic measurements were recorded before and after 3 months of treatment in both groups. Baseline thrombus morphology and mobility of both groups were well matched and are summarized in Table 1.

**Fig. 2:**
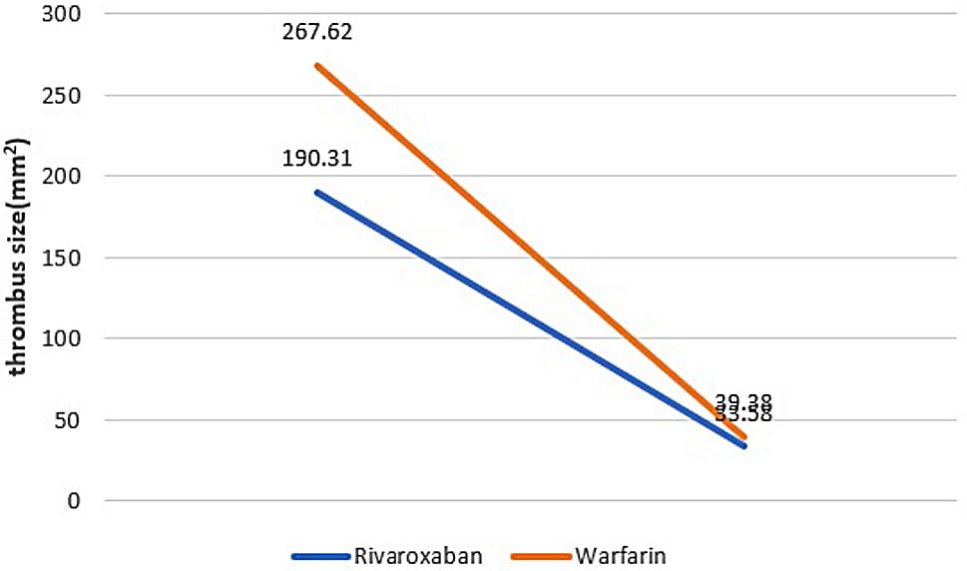
Comparison of thrombus size reduction between groups

Throughout the 3-month follow-up, both the warfarin and rivaroxaban groups showed a significant improvement (p-value < 0.05) in thrombus size. However, there was no statistically significant difference in the reduction of thrombus size between the rivaroxaban and Warfarin groups (p-value = 0.23, Table 3; Fig. 2). The mean thrombus size changes was − 133.11 ± 155.62 in the rivaroxaban group and − 228.23 ± 168.95 in the warfarin group, with the change being significantly higher in the warfarin group (Table 3).

**Table 3:** Comparison of thrombus size reduction between groups using nonparametric tests including Wilcoxon signed ranks test and Mann-Whitney test

Variables	Group	Before (Mean$\:\pm\:$SD)	After (Mean$\:\pm\:$SD	Z	*p*-value
Thrombus size (mm2**)**	**Rivaroxaban**	190.31$\:\pm\:$184.25	33.58$\:\pm\:$83.46	-1.68	0.000*
	Warfarin	267.62$\:\pm\:$223.98	39.38$\:\pm\:$76.19	-0.53	0.000**
	Z	-4.38	4.43		
	p-value	0.092^+^	0.590^++^		

*Comparing in rivaroxaban group using Wilcoxon Signed Ranks Test

**Comparing in warfarin group using Wilcoxon Signed Ranks Test

+Comparing before groups using Mann-Whitney Test

++Comparing after groups using Mann-Whitney Test

The non-resolved thrombi, comprising 6 in the rivaroxaban group and 8 in the warfarin group, underwent assessment using the paired samples McNemar test to observe changes in morphology and mobility before and 3 months after intervention. All immobile thrombi remained unchanged, while semi or hypermobile thrombi (2 in the rivaroxaban group and 7 in the warfarin group) transformed into immobile in both groups. However, the change from mobile to immobile was statistically significant only in the warfarin group (p-value = 0.016). Regarding morphology, the mural thrombi remained unchanged, and all round LVTs (2 in the rivaroxaban group and 4 in the warfarin group) changed into mural in both groups. The morphologic change was not significant in either group (Table 4).

**Table 4:** Comparing between groups

	Treatment group	Before	After		*p*-value
Mobility	Rivaroxaban		immobile	Semi or hypermobile	0.500
		Immobile	4	0	
		Semi or hypermobile	2	0	
	Warfarin		immobile	Semi or hypermobile	0.016
		Immobile	1	0	
		Semi or hypermobile	7	0	
Morphology	Rivaroxaban		Mural	Round	0.500
		Mural	4	0	
		Round	2	0	
			Mural	Round	0.125
		Mural	4	0	
		Round	4	0	

## Discussion

RCTs are widely regarded as the most accurate way of answering research questions. To the best of our knowledge, until recently, literature has been limited to observational studies and there is only one RCT to compare the efficacy and safety of rivaroxaban vs. Warfarin in post-ACS patients with LVT. Our study reveals several key findings. We observed no statistically significant difference in thrombus resolution, thrombus size improvement, risk of bleeding, or rehospitalization in patients treated with warfarin compared to those treated with rivaroxaban. Additionally, no embolic events, stroke, or Major Adverse Cardiovascular Events (MACE) were observed in either group. Among patients without complete resolution of thrombus, all mobile thrombi changed into immobile, and all round LVTs transformed into mural in both the rivaroxaban and warfarin groups. Furthermore, our results indicated that a greater proportion of subjects in the warfarin group achieved TTR ≥ 50%, correlating with a significantly higher rate of thrombus resolution. Overall, our findings suggest that rivaroxaban, is non-inferior to Warfarin for LVT management in ACS patients. These results are consistent with results seen in some prior observational studies and the recent clinical trial [12].

Although LVT can occur in settings other than infarction, its incidence is relatively high following an ST-elevation myocardial infarction (STEMI), and the medical management of LVT is very challenging due to its high risk of embolization and ischemic stroke. Anticoagulation therapy is one of the main parts of LVT management. Current guidelines (ESC, ACCF/AHA) primarily involve vitamin K antagonist (warfarin) for up to 3 months according to ACCF/AHA, with the Class IIa of recommendation and up to six months according to ESC, with the class IIa of recommendation, for treatment of post-ACS, LVT [6,16]. Concurrently, direct oral anticoagulants (DOACs) are advised for patients exhibiting poor control with warfarin. However, in recent years, the absence of a requirement for frequent monitoring, freedom from multiple drug interactions, and dietary restrictions increased the off-label use of DOACs among patients with LVT [17].

A recent meta-analysis on the safety and efficacy of DOAC vs. Warfarin in patients with LVT suggested that DOACs are non-inferior to Warfarin in terms of thrombus resolution, risk of bleeding, stroke and systemic embolization (SSE), and mortality which is congruent with our results [18]. Additionally, results from Jones et al. showed increased thrombus resolution with DOACs compared to warfarin [19].

Abdelnabi et al. recently conducted a non-LVT trial to compare efficacy and safety of rivaroxaban with Warfarin. They have prescribed 20 mg rivaroxaban daily for their subjects. Their patients were a combination of those with ACS and other cardiomyopathies. The results of this study showed that rivaroxaban was non-inferior to Warfarin with even faster resolution of thrombus in rivaroxaban group. They also reported more embolic events, stroke and major bleeding with Warfarin compared to rivaroxaban. In our study we showed that rivaroxaban is non-inferior to Warfarin for management of LVT and our results are consistent with this RCT [12].

LVT characteristics including mobility and morphology, which can be helpful in predicting the risk of embolization, were rarely discussed and assessed in previous studies. There are pieces of evidence that mural thrombus tends to be less mobile and has lower risk of embolization compared to round or protuberant thrombus [14,15]. In our study all of the round thrombi, those without complete resolution, transformed into mural thrombus in both rivaroxaban and warfarin groups.

In terms of thrombus size, a significant size reduction was detected in both warfarin and rivaroxaban groups with no statistically significant difference between groups. On the other hand, analysis of the difference in thrombus size changes was significantly higher in the warfarin group compared to the rivaroxaban group, and we think that the non-significantly greater baseline thrombus size in the warfarin group seems to be the reason.

Jugdutt et al. reported the occurrence of embolic events is associated with increased mobility of LVT in patients with acute MI [20]. Also, Oh et al. showed that thrombus mobility is the most important predictor of thrombus resolution and the first-ranked discriminator for embolic events [14]. Results from multiple studies and a recent meta-analysis performed by Dalia et al. found that there is no statistically significant difference in risk of stroke or systemic embolization (SSE) between Warfarin and DOACs, including rivaroxaban [18]. In our study all of the mobile LVTs, changed into immobile after intervention in both warfarin and rivaroxaban groups and the mobility change was statistically significant only in the warfarin group. The patients with resolved thrombus were not included in the pre and post-test analysis of mobility and morphology (because of the lack of data after thrombus resolution); So, because of the small number of patients in this analysis, it seems that we cannot conclude that Warfarin is more efficient than rivaroxaban in turning mobile LVT into immobile.

Although the results of our study support previous literature on the use of DOACs for the treatment of LVT, our study may have some limitations. The relatively small sample size may lead to low statistical power. Future studies can overcome this limitation by increasing their sample sizes. Also, another limitation is its single-center design, which may impact the generalizability of the findings. Future studies conducted across multiple centers are warranted to validate and extend our observations.

We also suggest to measuring the thrombus characteristics by more novel modalities including three-dimensional echocardiography, contrast enhanced TTE, or cardiac magnetic resonance imaging for future studies.

## Conclusion

In conclusion, in this randomized controlled trial we showed that rivaroxaban was non-inferior to Warfarin in thrombus resolution rate, thrombus size improvement, risk of bleeding, and rehospitalization rate. These results demonstrate that rivaroxaban may be an alternative option to Warfarin for the treatment of LV thrombus.

## Data Availability

No datasets were generated or analysed during the current study.
